# Rapidly-Developing Pleural Effusion: Explosive Pleuritis Caused by Group A Streptococcal Infection

**DOI:** 10.7759/cureus.26968

**Published:** 2022-07-18

**Authors:** Haris Asif, Mateus Fernandes, Allen Gorbonos, Arshan A Khan, Nader Ishak Gabra, Lucia Palladino

**Affiliations:** 1 Internal Medicine, Woodhull Medical Center, New York City, USA; 2 Internal Medicine, Ascension St. John Hospital, Grosse Pointe, USA; 3 Pulmonary and Critical Care, Woodhull Medical Center, New York City, USA; 4 Critical Care, Woodhull Medical Center, New York City, USA

**Keywords:** community acquired pneumonia, exudative pleural effusion, streptococcus pyogenes infection, rapid pleural effusion, explosive pleuritis

## Abstract

Community-acquired pneumonia is a leading cause of death from infectious diseases globally. Parapneumonic effusion is one of the most common complications of community-acquired pneumonia. As the infection progresses within the pleural space, loculation and empyema may develop. In rare cases, the parapneumonic effusions can progress significantly within 24 hours, which has been described as explosive pleuritis and may confer additional morbidity. Group A *Streptococcus* is the leading causative microorganism, which in itself has higher rates of parapneumonic effusions. We describe the case of a 30-year-old-female with a past medical history of asthma who presented to the emergency department with a sore throat, cough, and runny nose and was discharged on the same day after treatment of asthma exacerbation with upper respiratory tract infection. She re-presented within 24 hours with shortness of breath and right-sided pleuritic chest pain. Chest x-ray showed a new, large right-sided pleural effusion for which pleural fluid culture grew group A *Streptococcus*. She ultimately had prolonged hospitalization, requiring chest tube placement, and video-assisted thoracoscopic surgery (VATS). VATS was unsuccessful and she was treated with long-term antibiotics. This case demonstrates the dramatic evolution of explosive pleuritis and highlights the typical challenges encountered in these cases.

## Introduction

Community-acquired pneumonia (CAP) is a leading cause of morbidity and mortality in the United States, accounting for 4.5 million emergency room or outpatient visits annually [1]. CAP may be complicated by parapneumonic effusion (PPE) in 20-40% of cases [2,3]. In rare cases, there is a dramatic worsening of PPE within 24 hours, which has been described as “explosive pleuritis”. Explosive pleuritis is a rare and underreported diagnosis, associated with high morbidity and mortality. There is no widely accepted definition, but cases are usually distinguished by a significant, dramatic increase in the size of a pleural effusion within 24 hours of presentation [4]. In case reports, explosive pleuritis is commonly attributed to Group A beta-hemolytic *Streptococcus *(GAS). GAS is a gram-positive bacterium that frequently causes pharyngotonsillitis and infections of the skin and soft tissues but may also infiltrate the lung by inhalation or micro-aspiration [5,6]. Clinical presentation is similar to pneumococcal pneumonia with fever, pleuritic chest pain, dyspnea, and cough [4]. Patients with GAS may have worsened mortality and higher rates of pleural effusions. When explosive pleuritis occurs as a complication, empyema commonly develops requiring surgical management. We describe a case of explosive pleuritis and demonstrate the dramatic progression seen on imaging. 

## Case presentation

**Figure 1:**
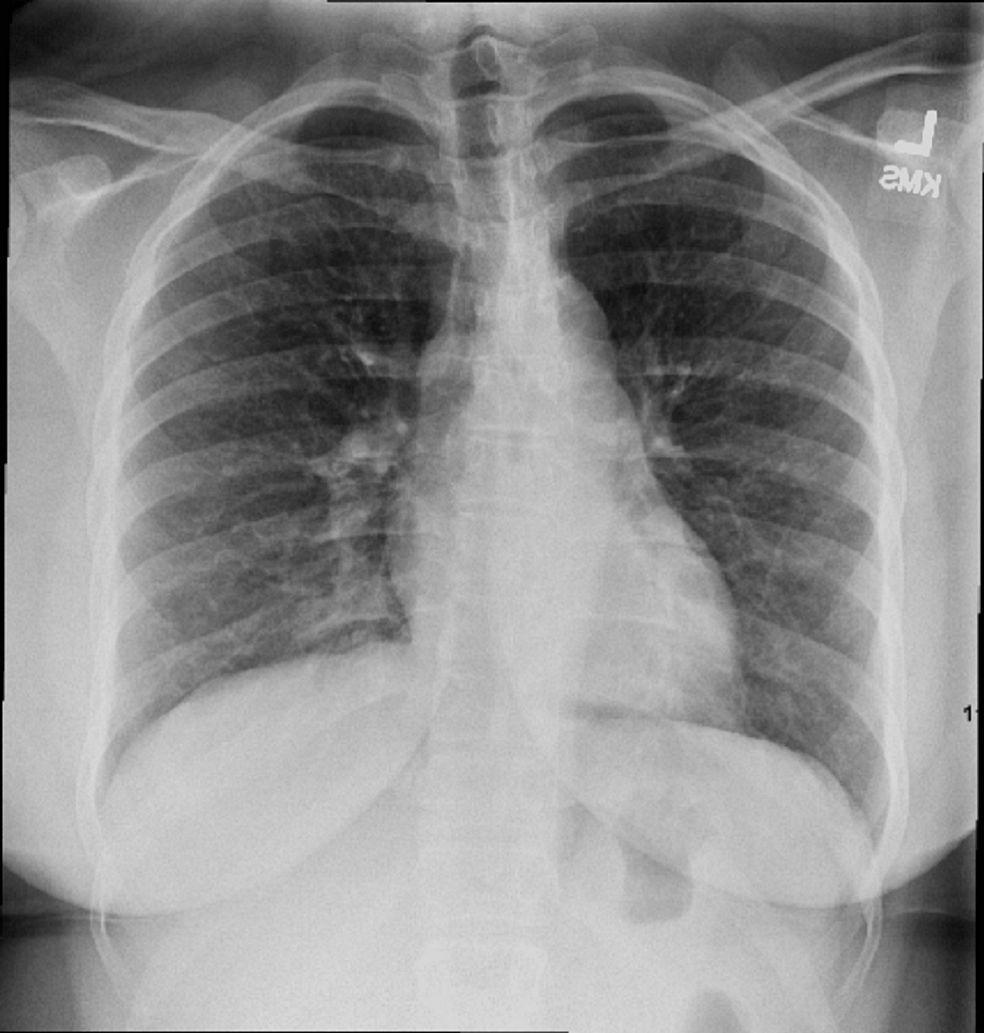
Chest x-ray on initial presentation, showing no infiltrate or any other significant changes

A 30-year-old female with a past medical history of asthma presented to the emergency department (ED with subjective fever, chills, body aches, and chest tightness. Patient reported sore throat, cough, and runny nose one day prior to symptom onset, which responded to nebulizer treatment at home. She denied any sick contacts or recent travels. She attributed her symptoms to asthma. On physical exam, lung auscultation revealed bilateral wheezing. Rhinorrhea was noted on exam. Her vital signs were within normal limits and without fever. Chest x-ray at that time showed no infiltrates and was otherwise unremarkable (Figure 1). Complete blood count was within normal limits. She was assessed as having an asthma exacerbation and was discharged from the ED with Ipratropium bromide/albuterol nebulization and prednisone. She presented to the ED less than 24 hours later with new right-sided pleuritic chest pain, worsening shortness of breath, subjective fevers, chills, and cough with green phlegm. She admitted drinking half a bottle of whiskey daily. Vital signs at the time were significant for blood pressure of 94/52 mmHg, heart rate of 163 beats per minute, respiratory rate 23 breaths per minute, oxygen saturation of 93% on room air, and temperature of 98.1 °F. Labs were significant for white cell count of 4.26, which increased to 12.62 by day four of admission. 

**Figure 2:**
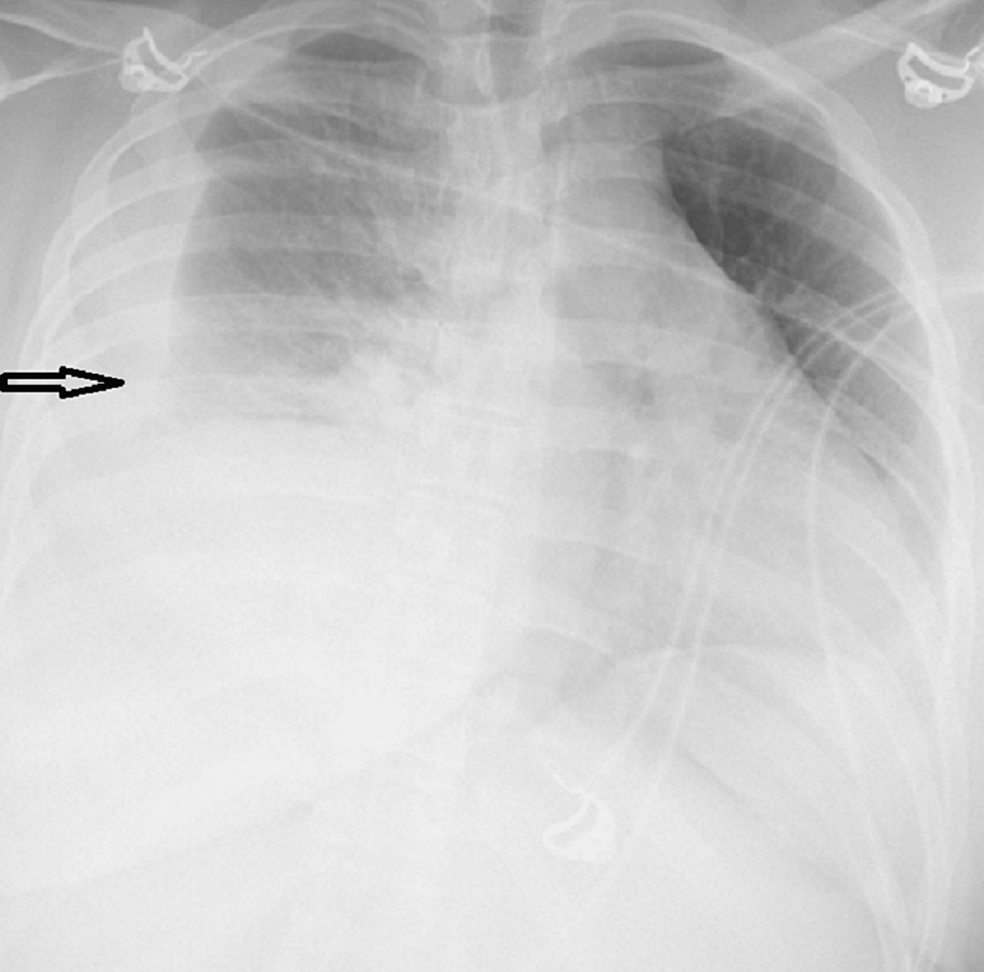
Chest x-ray done within 24 hours of initial presentation showing large right-sided pleural effusion (arrow)

**Figure 3:**
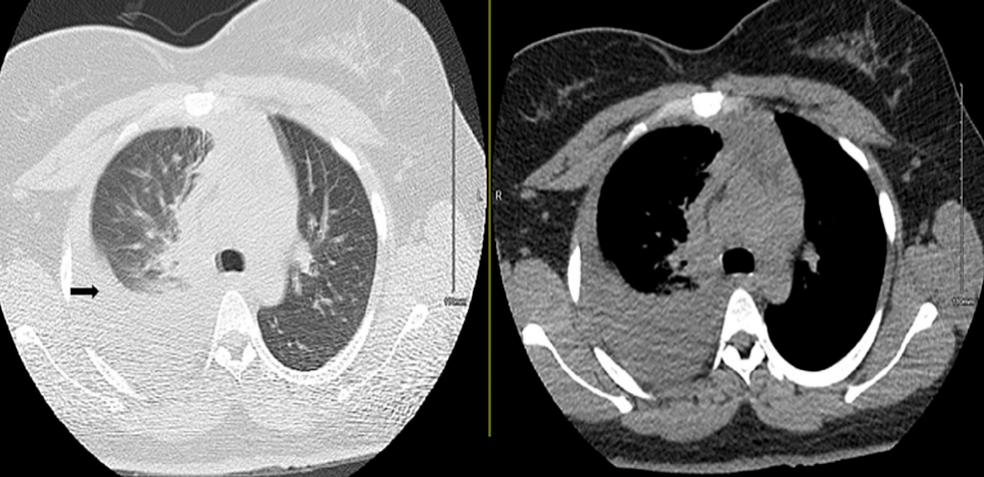
CT scan of chest done on the same day within 24 hours of initial presentation consistent with large right-sided pleural effusion (black arrow) CT: computerized tomography

Repeat chest x-ray showed a new, large right-sided pleural effusion (Figure 2). Computed tomography (CT) scan of the chest was done, which further defined a large right-sided pleural effusion (Figure 3). Patient was admitted to the step-down unit for further management of complicated parapneumonic effusion. A right-sided chest tube was placed, and pleural fluid was sent for analysis. Antibiotics were initiated, with a regimen comprising ceftriaxone and azithromycin. Pleural fluid analysis (Table 1) was consistent with an exudative pleural effusion.

**Table 1:** Results of pleural fluid analysis LDH: lactate dehydrogenase

Pleural Fluid Test	Result
pH	5.1
Protein	5.6 g/dL
Glucose	<20 mg/dL
LDH	7915 U/L
Color	Serosanguinous
White Cell Count	240, 700/uL

**Figure 4:**
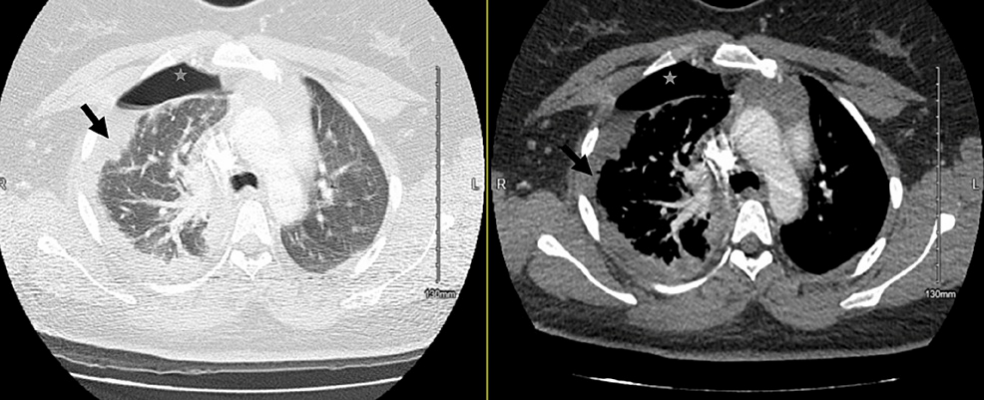
CT scan of chest showing right-sided loculated hydropneumothorax (grey star) and right-sided consolidation (black arrow) CT: computerized tomography

Blood and pleural fluid cultures, performed on day one, were positive for numerous *Streptococcus pyogenes*, for which sensitivities were not performed since GAS is usually universally susceptible to penicillin. Overall, these findings were consistent with explosive pleuritis resulting in empyema secondary to GAS. Antibiotics were narrowed to ceftriaxone; however, due to increasing leukocytosis (peaking at 33 on day seven from 4.26 on day one), on day five, clindamycin was added for antitoxin effects and ceftriaxone was discontinued. Repeat blood cultures on day five showed clearance of bacteremia. A repeat CT scan of the chest on the fourth day of hospitalization showed loculation and hydropneumothorax (Figure 4). After a minimal improvement in the patient’s clinical status and imaging, thoracic surgery was consulted for further management, and a decision was made to pursue video-assisted thoracoscopic surgery (VATS). VATS was attempted on day seven of hospitalization; however, the patient did not tolerate single lung ventilation and the procedure was aborted. To facilitate further drainage in lieu of VATS, two 32 Fr chest tubes were placed. Repeat imaging showed an improved appearance of consolidation and resolving effusion. Chest tubes were removed by day 10, and white cell count continued to decline, improving to 6.58 by day 21. She had no new fevers, blood cultures remained negative, and she completed eight days of clindamycin, ultimately transitioning to amoxicillin-clavulanate with at least six weeks duration planned.

## Discussion

Pneumonia may be complicated by parapneumonic effusion in 20-40% of cases with 2-3% further complicated by empyema [7]. A proposed definition for explosive pleuritis is a pleural effusion that dramatically progresses over 24 hours (4). It was first described by Braman and Donat in 1986 as an effusion that developed within hours of admission. They presented two cases of explosive pleuritis caused by group A beta-hemolytic *Streptococcus* in the absence of bronchopneumonia [8]. Sharma and Marrie redefined the entity as the rapid development of pleural effusion involving more than 90% of the hemithorax within 24 hours causing compression of the pulmonary tissue and a mediastinal shift [9]. Interestingly, as in our case, the cases described by these authors were mostly young individuals without any major comorbidities and risk factors [10].

The etiology and pathogenesis of explosive pleuritis are not well understood. The initial authors describing it proposed that streptococcal infections may have a unique feature of blocking the peri bronchial and sub-pleural lymphatics with cellular and necrotic debris, which leads to rapid accumulation of pleural effusion [8]. A unique feature of GAS-induced pneumonia is the rapid accumulation of pleural effusion in up to 80% of cases compared to 10 % in pneumonia caused by other organisms [11]. Major organisms responsible for the occurrence of explosive pleuritis are gram-positive cocci (commonly *Streptococcus pneumonia*, *Streptococcus pyogenes* and other streptococci, staphylococci), gram negative cocci, *Neisseria meningitidis*, and *Moraxella catarrhalis*. Gram-negative rods as well as mycobacteria, fungi, *Mycoplasma*, *Chlamydia*, and *Rickettsia* may also be included [9].

The clinical features of explosive pleuritis are similar to other causes of pleural effusion. It commonly includes fever, dyspnea, cough, pleuritic chest pain, and hemoptysis [9]. Laboratory features include elevated inflammatory markers and positive blood cultures in almost 40% of cases [12]. Pleural fluid analysis is essential for definitive diagnosis. Pleural fluid analysis is usually exudative by Light’s criteria (Pleural fluid protein concentration and LDH are often greater than 3 g/dl and 550 U, respectively). Cultures are positive from pleural fluid in the majority of cases. Chest x-ray would reveal pleural effusion, which may or may not be loculated. CT scan can aid in identifying the extent and nature of the massive pleuritis. In some cases, blood cultures, sputum cultures, and pleural fluid cultures might not reveal the causative organism due to administering the antibiotics at an early stage. The rapid progression of radiographic findings and quick deterioration of the patient’s clinical status should warrant a diagnosis of exclusive pleuritis [9].

Management of explosive pleuritis starts with prompt initiation of antibiotics with group A streptococcal coverage including penicillin plus the use of clindamycin for toxin control [13]. Early thoracentesis is essential for diagnostic and therapeutic purposes, which should be followed by prompt drainage via a chest tube [10]. Imaging of the chest should be performed following chest tube placement for assessing the accurate placement of the chest tube, assess for complications, and to monitor response. Further management may be guided by signs and symptoms, radiographic progression, and drainage volume. Deterioration in radiographic imaging and patient’s clinical status should prompt the clinician in considering surgical drainage through thoracotomy or VATS for decortication and drainage. In our case, a CT scan on day four of hospitalization showed a loculated right-sided hydropneumothorax and right-sided consolidation, which prompted further management with VATS. Unfortunately, the patient could not tolerate single lung ventilation and the procedure was aborted. In patients who are not candidates for surgery (congestive heart failure, chronic lung disease, peripheral vascular disease, metastatic cancer, diabetes with complications, coagulopathy, anemia of chronic disease, and sepsis) or do not want surgery, intrapleural tissue plasminogen activator with DNase can be considered [14].

## Conclusions

Explosive pleuritis is a rare complication of parapneumonic effusion, distinguished by a dramatic worsening of pleural effusion within 24 hours or presentation. Group A *Streptococcus* is the leading causes of explosive pleuritis. Explosive pleuritis may confer higher morbidity and mortality, thus early recognition and treatment is imperative. Thoracentesis and pleural fluid analysis are essential for diagnosis. Most cases require chest tube drainage for resolution. In unresolved cases surgical approach such as thoracotomy and VATS procedure should be considered.

## References

[1] Ticona JH, Zaccone VM, McFarlane IM (2021). Community-acquired pneumonia: a focused review. Am J Med Case Rep.

[2] Light RW (2006). Parapneumonic effusions and empyema. Proc Am Thorac Soc.

[3] Grijalva CG, Zhu Y, Nuorti JP, Griffin MR (2011). Emergence of parapneumonic empyema in the USA. Thorax.

[4] Zoumot Z, Wahla AS, Farha S (2019). Rapidly progressive pleural effusion. Cleve Clin J Med.

[5] Basiliere JL, Bistrong HW, Spence WF (1968). Streptococcal pneumonia. Recent outbreaks in military recruit populations. Am J Med.

[6] Keefer CS, Rantz LA, Rammelkamp CH (1940). Hemolytic streptococcal pneumonia and empyema; a study of 55 cases with special reference to treatment. Trans Am Clin Climatol Assoc.

[7] Weese WC, Shindler ER, Smith IM, Rabinovich S (1973). Empyema of the thorax then and now. A study of 122 cases over four decades. Arch Intern Med.

[8] Braman SS, Donat WE Explosive pleuritis. Manifestation of group A beta-hemolytic streptococcal infection. Am J Med.

[9] Sharma JK, Marrie TJ (2001). Explosive pleuritis. Can J Infect Dis.

[10] Al-Mashat M, Moudgal V, Hopper JA (2015). Explosive pleurisy related to group A streptococcal infection: a case report and literature review. Pulm Res Respir Med Open J.

[11] McMurray JJ, Fraser DM, Brogan O (1987). Fatal Streptococcus pyogenes pneumonia. J R Soc Med.

[12] Kumar S, Sharath Babu NM, Kaushik M, Verma BS, Kaushal SS (2012). Explosive pleuritis. J Allied Health Sci Pract.

[13] Low DE (2013). Toxic shock syndrome: major advances in pathogenesis, but not treatment. Crit Care Clin.

[14] Semenkovich TR, Olsen MA, Puri V, Meyers BF, Kozower BD (2018). Current state of empyema management. Ann Thorac Surg.

